# The Synergistic Effect of N2 and N7 Modifications on the Inhibitory Efficacy of mRNA Cap Analogues

**DOI:** 10.3390/ph17050632

**Published:** 2024-05-14

**Authors:** Karol Kurpiejewski, Karolina Piecyk, Maciej Lukaszewicz, Karol Kamel, Kazimierz Chmurski, Sebastian Kmiecik, Marzena Jankowska-Anyszka

**Affiliations:** 1 Faculty of Chemistry, University of Warsaw, 02-093 Warsaw, Poland; k.kurpiejewski@uw.edu.pl (K.K.); ke.piecyk@uw.edu.pl (K.P.); chmurkaz@chem.uw.edu.pl (K.C.); 2 Division of Biophysics, Institute of Experimental Physics, University of Warsaw, 02-093 Warsaw, Poland; maciej.lukaszewicz@fuw.edu.pl; 3 Institute of Bioorganic Chemistry, Polish Academy of Sciences, 61-704 Poznan, Poland; karol.kamel@gmail.com; 4 Biological and Chemical Research Centre, Faculty of Chemistry, University of Warsaw, 02-089 Warsaw, Poland; sekmi@chem.uw.edu.pl

**Keywords:** nucleotide, cap analogues, mRNA, cap-dependent translation, inhibitor

## Abstract

In the fight against cancer, researchers have turned their attention to the eukaryotic initiation factor eIF4E, a protein whose increased level is strongly correlated with the development and progression of various types of cancer. Among the numerous strategies devised to tackle eIF4E overexpression, the use of 5′ end mRNA cap analogues has emerged as a promising approach. Here, we present new candidates as potent m^7^GMP analogues for inhibiting translation and interfacing with eIF4E. By employing an appropriate strategy, we synthesized doubly modified mono- and dinucleotide cap analogues, introducing simultaneous substituents at both the N7 and N2 positions of the guanine ring. This approach was identified as an effective and promising combination. Our findings reveal that these dual modifications increase the potency of the dinucleotide analogue, marking a significant advancement in the development of cancer therapeutics targeting the eIF4E pathway.

## 1. Introduction

**Figure 1:**
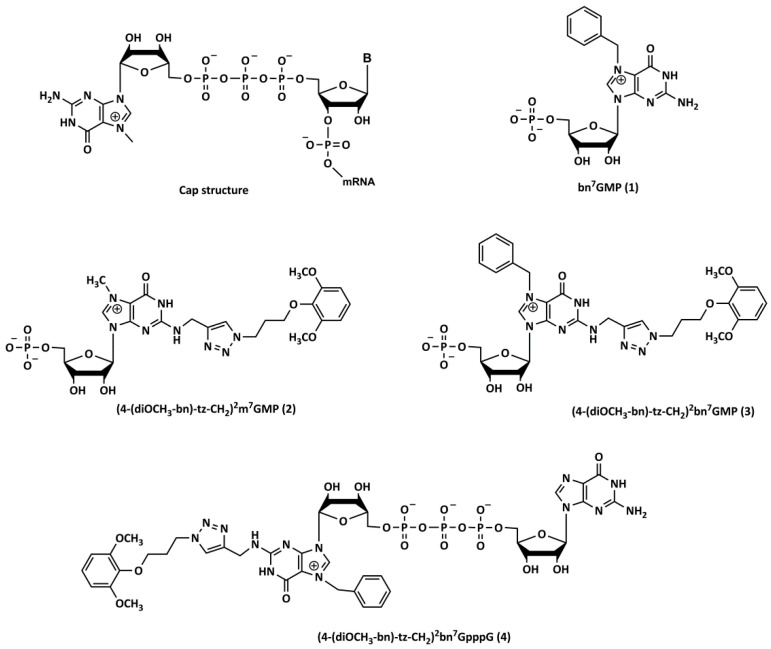
The chemical structure of an mRNA cap and its analogues.

**Figure 2:**
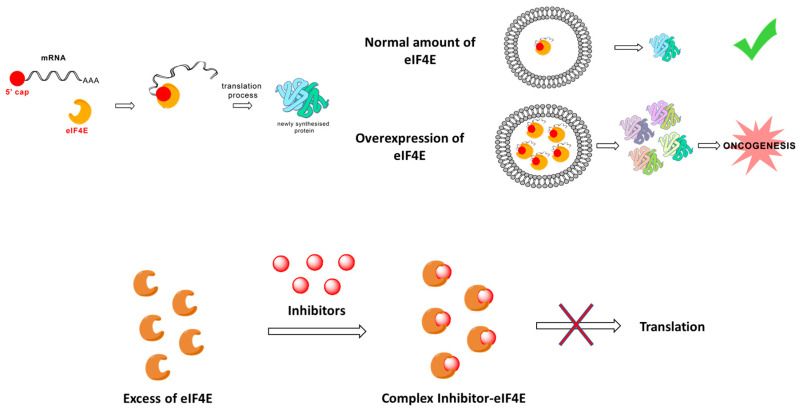
Idea of translation inhibition by cap analogues.

All eukaryotic RNAs synthesized by RNA polymerase II undergo a characteristic modification at their 5′ ends by the addition of a cap [1]. A key structural feature of the 5′ end of mRNA is a 7-methylguanosine (m^7^G) linked to the transcript residue via a 5′,5′-triphosphate bridge, which is atypical for nucleic acids (Figure 1, structure shown in the top row, left side). In general, the cap performs its biological functions by interacting with various proteins that specifically recognize its structure, e.g., eukaryotic initiation factor 4E (eIF4E) binds the cap structure during translation initiation [2,3]. The intracellular level of eIF4E is low, and changes in its level fall into one of the mechanisms that allows gene expression to be regulated at the translational level [4]. The idea of using cap analogues for therapeutic purposes came with reports that an elevated level of eIF4E is associated with tumor formation and progression in human malignancies and cancers of the breast, colon, bladder, prostate, lung, head, and neck (Figure 2, the top row) [5,6,7]. Among various strategies to counteract eIF4E overexpression, methods based on cap analogues have emerged as particularly effective, according to recent reports [8]. By binding to eIF4E, synthetic cap analogues of the 5′ end of mRNA prevent the association of eIF4E with the mRNA cap, thereby inhibiting translation (Figure 2, the bottom row).

X-ray crystallography and multidimensional nuclear magnetic resonance [9,10,11], utilizing m^7^GDP, m^7^GTP, and m^7^GpppG/A as representative compounds of the functional 5′ end of mRNA in complex with mammalian eIF4E, along with complementary biophysical experiments [10,12,13], showed that the m^7^G-containing fragment occupies the binding pocket between two tryptophan residues, Trp56 and Trp102, forming sandwich cation-π stacking. In addition, the m^7^G moiety forms three hydrogen bonds: two with Glu103 via an exocyclic amino group and one with Trp102 involving an oxygen atom of guanine. The triphosphate chain forms salt bridges, and direct or indirect (via water) hydrogen bonds with the indole rings of Trp102 and Trp166, as well as with the side chains of Arg112, Arg157, and Lys162.

Numerous modified cap analogues have been tested so far as inhibitors of cap-dependent translation in vitro that compete with mRNA for the eIF4E binding site [14,15,16,17,18]. Unfortunately, due to their charged nature, the use of such analogues in vivo is strictly limited. One effective method to overcome this problem is to create modified monophosphate pro-nucleotides (ProTide). First in this class was 4Ei-1, a prodrug that is transformed intracellularly into the functional 7-benzylguanosine monophosphate (bn^7^ GMP, **1**, Figure 1) [19]. Bn^7^GMP is the flagship example of cap analogues that have been used to prepare various types of pro-drugs [20,21]. Since generally, monophosphate cap analogues exhibit poor translational inhibitory properties, an additional modification of these structures is required to compensate for this deficit and a modification of the N7 guanosine position is one of the chosen sites. Brown and co-workers sought to clarify by crystallographic measurements why monophosphate analogues with the large benzyl group at the N7-guanosine position show higher affinity for the 4E-initiating factor than expected [22]. These studies led to the conclusion that, during complex formation between the protein and bn^7^GMP, a conformational change occurs in the binding pocket involving a 180° rotation of the indole ring of Trp102. This conformational change allows the bulky benzyl group to easily fit into the binding pocket. Furthermore, the rotation of Trp102 does not disrupt any pre-existing interactions, including the π-stacking interaction between the purine ring and Trp56 and Trp102, or the formation of hydrogen bonds. When comparing m^7^GMP with bn^7^GMP, it appears that the affinity of the analogue having an aromatic ring for eIF4E increases due to the additional interactions.

Another type of modification that makes the monophosphate a potent inhibitor is those introduced at the N2 position of m^7^G, which have also been verified and tested in the ProTide form [20]. Studies have shown that a single substitution at the N2 position of the m^7^G monophosphate leads to increased translational inhibition [23]. Among those N2-modified cap analogues with good inhibitory properties are compounds containing a triazole moiety, in addition to the phenyl ring [24]. One such compound is shown in Figure 1 (structure labelled **2**). An evaluation of the ability of such triazole-containing monophosphates to inhibit cap-dependent translation in a cell-free translation system showed that such analogues are as effective as translation inhibitors such as m^7^GpppG and even m^7^GTP. It was also observed that the presence of a triazole ring at the N2 position of guanine is sufficient to make a simple 5′-monophosphate analogue of the cap a potent inhibitor of cap-dependent translation.

Taking into account the positive effect of both modifications discussed above on the ability of monophosphates to inhibit translation processes, we decided to obtain a new monophosphate analogue simultaneously modified at the N2 and N7 positions of guanosine. Considering the results obtained for the variously modified analogues, we selected the substituents that showed the best properties in regard to translation inhibition and binding to the eIF4E factor when present at the N7 or N2 position as a single modification. Therefore, we chose to introduce a benzyl substituent at the N7 position and a modified triazole substituent at the N2 position (Figure 1, compounds **3** and **4**). The newly designed monophosphate analogue was first subjected to molecular modeling to predict the mode of their interaction with eIF4E. In the next step, the proposed monophosphate analogue and its corresponding dinucleotide were obtained in a multi-step chemical synthesis and finally tested as potential inhibitors.

## 2. Results and Discussion

To test the feasibility of using and assessing the potential of double-modified analogues of the 5′ end structure of mRNAs as translation inhibitors, the results obtained for analogues containing a single modification exclusively at the N7 position of the guanosine-5′-phosphate position and at the N2 position of 7-methylguanosine-5′- phosphate position were analyzed. On this basis, two substituents were selected for their superior inhibitory properties compared to m^7^GMP: a benzyl substituent at the N7 position (Figure 1, compound **1**) and a (1-(3-(2,6-dimethoxyphenoxy)propyl)-1H-1,2,3-triazol-4-yl)methyl substituent at the N2 position (Figure 1, compound **2**). 

### 2.1. Molecular Docking Studies

To determine whether the designed monophosphate cap analogue with two modifications at the N2 and N7 positions can bind to eIF4E and thus act as an inhibitor of the translation process, we initially performed molecular modeling using the human eIF4E protein structure. This approach was particularly important because, unlike for the bn^7^GMP [22], no crystallographic structure of mammalian eIF4E in complex with any N2-modified analogues is known.

**Figure 3:**
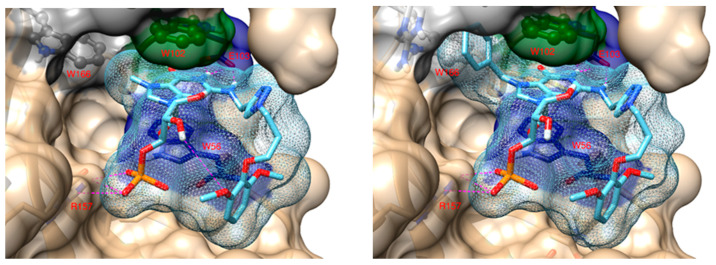
(**left**) The docked pose of compound **2** with an amino acid interaction pattern. Hydrogen bonding (in magenta) with Trp56, Glu103, and Arg157 and π-electron stacking of guanosine ring with Trp56 and Trp102. The N7 methyl group forms van der Waals contact with Trp166. (**right**) The binding mode of compound **3** with an amino acid interaction pattern. The hydrophobic cavity is filled with a benzyl substituent, which provides much stronger anchoring for the compound.

Therefore, we opted to perform initial molecular modeling for the analogue with a substituent solely at the N2 position. For analogue **2** (Figure 3), seven out of ten docking runs yielded the model depicted in Figure 3 left [25,26]. In our model, the guanosine ring of analogue **2** aligns similarly to m7GTP when modeled with the eIF4E protein. The purine ring engages in π-electron stacking with Trp56 and Trp102 and forms van der Waals contacts with Trp166. Furthermore, hydrogen bonding involves the protons of the N1 and N2 nitrogen atoms with Glu103. Interestingly, the phosphate group interacts with Arg157. Despite the structural flexibility between the purine base, sugar, and phosphate group, which could allow interactions with Lys162, our model suggests a preferred alignment. Notably, the triazole group, while not directly interacting with the protein, enhances the compound’s solubility due to its hydrophilic nature, facilitating accessibility to the protein. Its structural length enables anchoring to the amide proton of Trp56 through the methoxy group.

Additionally, we developed a model for compound **3** (Figure 3), identified as a new potential translation inhibitor. We anticipate this analogue, featuring an N2-triazole and an additional N7 modification, to achieve greater cap-dependent inhibition by fitting into the eIF4E’s hydrophobic binding pocket. This approach mirrors that of Y. Jia et al. [15], who aimed to enhance ligand binding by enlarging the hydrophobic pocket surrounding Trp102, Arg112, Trp166, His200, and Thr203 for accommodating the N7 substituent. Furthermore, our findings show that the triazole substituent at the N2 position keeps the protein conformation unchanged, similarly to compound **2**.

Based on the promising model of this new potential translation inhibitor, synthesizing the designed compound and assessing its biological properties became a justified next step.

### 2.2. Chemical Synthesis

**Scheme 1:**
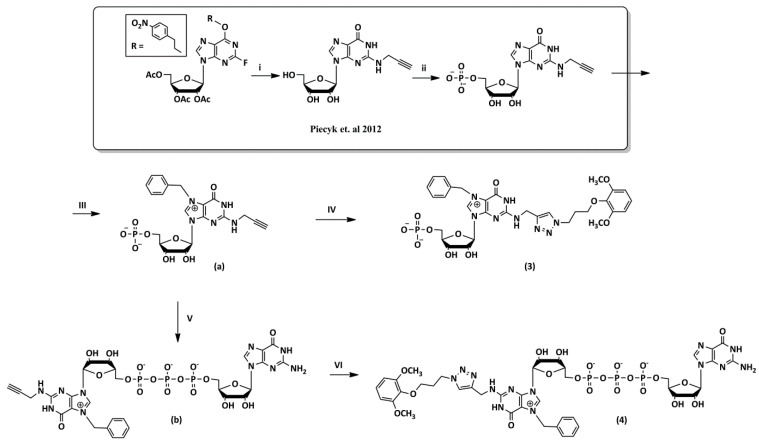
A schematic of the synthesis strategy to obtain newly designed cap analogues. The initial steps of synthesis were performed according to the previously described procedure [23]. Reagents: (I) propargylamine/DMSO, (II) POCl_3_/TMP, (III) benzyl bromide/DMSO, (IV) 2-(3-azidopropoxy)-1,3-dimethoxybenzene/CuSO_4_/sodium ascorbate/DMSO, (V) im-GDP/ZnCl_2_/DMF, and (VI) 2-(3-azidopropoxy)-1,3-dimethoxybenzene)/CuSO_4_/sodium ascorbate/H_2_O/DMSO.

The synthesis of compounds **3** and **4** involved a multi-step reaction, starting with fully protected 2-fluoro inosine as the initial material for both compounds. This was transformed into N2-propargyl guanosine through aromatic substitution followed by selective deprotection (Scheme 1). Subsequently, the N2-modified guanosine derivative was 5′-phosphorylated with phosphine oxide trichloride in trimethyl phosphate at 4 °C. Purification of the analogue was performed by ion exchange chromatography on DEAE-Sephadex A-25. The resulting monophosphate was further modified by alkylation of the N7 position of the guanine ring with benzyl bromide in DMSO at RT; the reaction product was isolated by ion exchange chromatography. Finally, the mononucleotide product **3** was synthesized by a click reaction with the 2-(3-azidopropoxy)-1,3-dimethoxybenzene, followed by isolation and purification via RP HPLC. The necessity to apply the proposed strategy was dictated by the fact that there is a possibility of substitution of the benzyl substituent into the triazole moiety. In order to avoid the formation of side products, this order of reaction is favored.

While mononucleotide cap analogues have been explored as inhibitors, there is growing interest in dinucleotide analogues for similar application [14,27]. Accordingly, we decided to investigate a dinucleotide analogue of the cap in the form of a new double-modified monophosphate analogue bound via a 5′-5′ triphosphate bridge to guanosine (Figure 1, compound **4**). This extended structure not only has potential in RNA engineering, as recent studies have shown [28], but also offers promising avenues for designing new translation inhibitors Moreover, it can show better solubility in water, which will allow testing in an environment without the addition of organic solvents. To obtain the dinucleotide product **4**, the synthesis pathway chosen involved initiating a coupling reaction between compound (**a**) and the imidazole derivative of GDP (im-GDP), followed by a ‘click’ reaction with 2-(3-azidopropoxy)-1,3-dimethoxybenzene (Scheme 1). Alternatively, conducting the reaction directly with compound **3** (obtained previously) to yield dinucleotide **4** through a coupling reaction with im-GDP was a possibility. However, considering the relatively low yields and the time required for purification (solely via HPLC) of compound **3**, this approach was deemed unviable for obtaining the final product (**4**). Therefore, in a first step, coupling reactions catalyzed by anhydrous zinc chloride were carried out between the im-GDP and the obtained bn^7^GMP-modified propargyl at the N2 position under anhydrous conditions. In the next step, the obtained dinucleotide was used in a click reaction with 2-(3-azidopropoxy)-1,3-dimethoxybenzene). The final product was isolated by ion exchange chromatography and further purified by RP HPLC.

### 2.3. Inhibitory Properties

**Figure 4:**
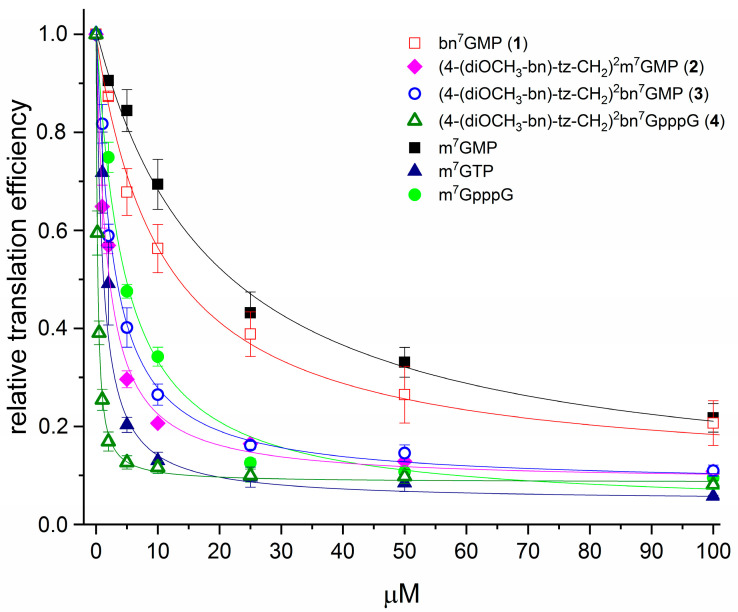
The inhibition of translation in rabbit reticulocyte lysate (RRL) with the analyzed compounds. The presented data points correspond to the mean of 3–6 independent experiments (±SE). The fitting curves used in the IC_50_ value determination are shown.

To estimate the ability of the newly synthesized double-modified nucleotides to inhibit cap-dependent translation, the extracellular translation system from rabbit reticulocytes lysate (RRL) was used according to previously described procedures [24]. This procedure was implemented because it mimics the competition between the cap analogue and the mRNA in the all-natural system. Briefly, experiments were performed in a micrococcal nuclease-treated RRL using an in vitro-transcribed, ARCA-capped, β-globin 5′ UTR containing mRNA encoding firefly luciferase to allow for the determination of protein synthesis by luminometry. In the experiments, three standard cap analogues served as controls: m^7^GTP and m^7^GpppG were employed as positive controls due to their established efficacy as translation inhibitors in various cell-free systems [14,29], whereas m^7^GMP, known for its minimal or nonexistent inhibitory capacity, was a negative control [14]. As the resulting monophosphate compound **3** is only partially soluble in water, biological tests were performed in a solution containing a small amount of dimethyl sulfoxide (DMSO). Initially, the compounds were prepared in an aqueous solution containing 10% DMSO, and inhibition was measured at a final concentration of 1% DMSO. Incubations were conducted at several series of inhibitor concentrations in three independent measurement series (Figure 4). The results were obtained by bioluminescence measurement, allowing for the determination of IC_50_ values for all compounds tested (Table 1). 

Since the study was performed in a 1% DMSO solution due to the poor solubility of the new monophosphate analogue, the measurements for the standard compounds were also carried out in the same conditions. We also determined the IC_50_ for bn^7^GMP (**1**) and monophosphate **2** under the same conditions to assess the effect of the presence of both substituents in the new compound **3**. The presence of 1% DMSO in the reticulocyte lysate translation mixture resulted in a more pronounced inhibition of the translation for m^7^GMP, m^7^GTP, and m^7^GpppG compared to the results obtained for their water solutions (similarly to the data obtained in the presence of another organic solvent, DMF, which was used previously in inhibition studies of isoxazole analogues [30]). The previously determined IC_50_ value for compound **2** in water was 2.00 ± 0.10 μM [24], while in DMSO solution, it had an IC_50_ value of 1.80 ± 0.21 μM, indicating little effect of the addition of an organic solvent. On the other hand, it is a slightly weaker inhibitor than m^7^GTP, although in pure water, both compounds had similar IC_50_ values. This suggests a differential effect of a small addition of DMSO on the inhibition measurement for compounds with a substituent in the N7 position and compounds with a substituent in the N2 position of guanosine. This may be related to the different positions of the two substituents when interacting with eIF4E. As shown by molecular modeling, the benzyl substituent is hidden in the hydrophobic binding pocket of the protein while the substituent in the N2 position is solvent-exposed. 

In the case of bn^7^GMP (**1**), our results indicate that it has significantly worse inhibitory properties compared to m^7^GTP and around two times better ones than m^7^GMP. The results presented so far for this compound have been obtained using different measurement methods in different in vitro systems, leading to a large discrepancy in IC_50_ values. In the work of Cai et al. [14] and Brown et al. [22], bn^7^GMP showed approximately three times stronger inhibitory properties compared to m^7^GMP, in the studies of Jia et al. [15] and Li et al. [19]. These properties were even more pronounced (5.7- and 8-fold stronger effects, respectively). In a 2012 paper [16], inhibition experiments were performed under similar conditions to ours, i.e., in a 1% DMSO solution. The authors showed that the inhibitory properties of bn^7^GMP were 1.5 times better than those of m^7^GMP, which is in agreement with our results and indicates a slight positive effect of the benzyl compared to the methyl substituent at the N7 position of guanosine.

The newly synthesized monophosphate analogue **3**, which features both a benzyl substituent at the N7 position and an aromatic substituent at the N2 position, effectively inhibits the translation process. However, its inhibition capability is less pronounced than that of the analogue with only the N2 substituent (IC_50_ = 2.70 ± 0.30 μM versus 1.80 ± 0.21 μM). This differential efficacy may be attributed to the inherent properties of the compound, notably its lower solubility in pure water compared to compound **2**. This assumption is supported by the performance of dinucleotide **4**, which carries identical substituents at both the N2 and N7 positions of guanosine and demonstrates significantly higher inhibitory potency—almost 18-fold greater than standard m^7^GpppG (IC_50_ = 0.24 ± 0.02 μM versus 4.45 ± 0.61 μM). Furthermore, a comparison with previously tested dinucleotide analogues as translation inhibitors—bn^7^GpppG and m^7^GpppG analogues with the same aromatic substituent at the N2 position ((4-(diOCH_3_-bn)-tz-CH_2_)^2^m^7^GpppG) as compound **2**—reveals that these exhibited approximately 1.5 and 6.9 times more potent inhibition than the standard dinucleotide, respectively [27,28]. Notably, these experiments were conducted in pure water without the addition of DMSO, suggesting that for the dinucleotide analogue, the combined presence of substituents at both the N2 and N7 positions significantly enhances the inhibitory effectiveness beyond the impact of either substituent alone. 

### 2.4. Thermal Stabilization of eIF4E Analyzed by Differential Scanning Fluorimetry

**Figure 5:**
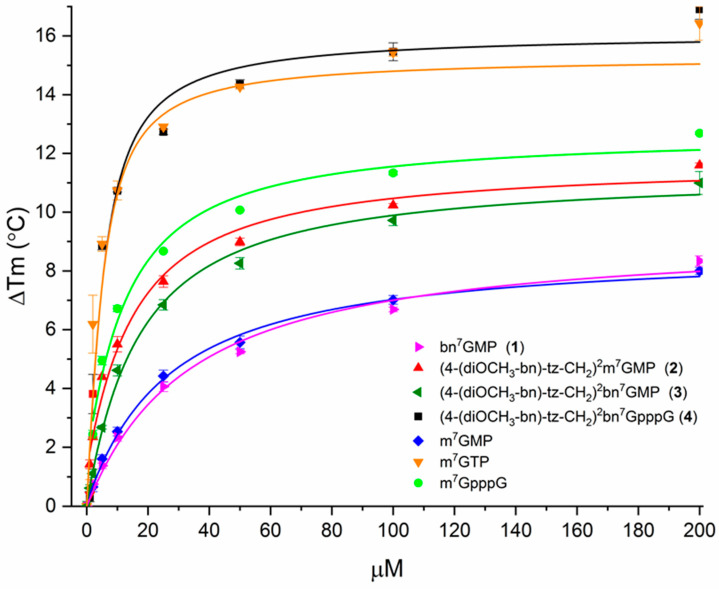
The thermal stabilization of heIF4E in response to the increasing concentration of the examined compounds. The solid lines correspond to the fitted curves of the single site ligand binding model used for the apparent affinity (appK) estimation (as described in 2014 [32]). The ΔTm values (±SE) correspond to three independent experiments.

Next, we analyzed the effect of analogues **3** and **4** on the thermal stabilization of the human eIF4E (heIF4E) translation initiation factor upon ligand binding using differential scanning fluorimetry (DSF) [31]. Similarly to the inhibition experiments described earlier, the tested compounds in 10% DMSO were used to give the 1% DMSO final concentration in DSF measurements. The melting temperature (Tm) of heIF4E alone (in the absence of a ligand) upon these experimental conditions was 39 °C (which is similar to the Tm value reported earlier for murine eIF4E) [28,30]. As shown in Figure 5, there is a gradual increase in the heIF4E melting temperature (Tm) in the presence of increasing concentrations of the tested and control compounds. The most prominent effect is seen for analogue **4**, which results in a 10-degree increase in the thermal stability of heIF4E (ΔTm ~10 °C) below a 10 μM final concentration, similar to the m^7^GTP control compound. Analogue **3** exhibited a stabilizing impact on heIF4E similar to that observed with the m^7^GpppG dinucleotide and analogue **2**, as demonstrated in Figure 5. 

The apparent affinity values (appK), calculated on the basis of the ΔTm change in heIF4E in response to an increasing concentration of the tested ligand, are shown in Table 2. Notably, m^7^GTP demonstrates approximately eight times higher affinity for heIF4E compared to m^7^GMP. This finding correlates well with the tenfold difference in the dissociation constant (Kd) of these compounds, as determined by a fluorescence polarization (FP) assay conducted under a similar condition of 1% DMSO, consistent with our DSF methodology [18]. Next, the appK values for m^7^GMP and bn^7^GMP (**1**) are in the same range (Table 2), and similar values of apparent Kd were reported for these two compounds based on the pyrene probe fluorescence intensity method (Kd,app for m^7^GMP and bn^7^GMP in μM range—37 μM and 25.8 μM, respectively) [21]. Importantly, the single modification introduced at the N2 position (analogue **2**) and the double modification introduced at the N2 and N7 positions (analogue **3**) increased the affinity to heIF4E by around two times in comparison to the unmodified mononucleotide, to the extent seen for m^7^GpppG. Extending analogue **3** into its dinucleotide counterpart (analogue **4**) further increased the binding affinity to heIF4E (around 7x in comparison to m^7^GMP), which suggests additional stabilizing interactions of phosphates in the triphosphate bridge (and/or guanosine) of this dinucleotide cap analogue with heIF4E.

## 3. Materials and Methods 

### 3.1. Molecular Modeling

A model of the eukaryotic translation initiation factor 4E (eIF4E) protein was prepared based of the 2v8x structure from the PDB bank. The addition of hydrogen atoms to the protein model was carried out in the UCSF Chimera program [25]. All the ionizable residues were protonated according to physiological pH. The molecular docking of the cap analogues was performed using the AutoDock Vina software [26]. The ligand was placed in a cuboid box of dimensions 34 × 29 × 36 Å, which allowed for efficient sampling of the ligand-accessible regions. The number of binding modes in the output was set to 20. Ten independent docking runs were performed.

### 3.2. Chemistry

General information: All used chemical reagents, the starting materials and m^7^GTP, were purchased in the highest available purity from commercial sources and were used without any further treatment. P-Imidazolide of 5′-diphosphate guanosine (im-GDP), m^7^GMP, m^7^GpppG, bn^7^GMP (1), N2-(prop-2-yn)-GMP, and compound **2** were synthesized based on previously described protocols [13,23,24]. A triethylammonium bicarbonate (TEAB) buffer was prepared by bubbling CO_2_ through an ice-cold aqueous solution of redistilled triethylamine. The intermediate nucleotides and the final product (**4**) were separated by ion-exchange chromatography on a DEAE-Sephadex A-25 (HCO_3_^−^ form) using a linear gradient of the TEAB buffer (pH 7.6). An HPLC analysis was performed on a Knauer instrument (Smartline System and ClarityChromPrep software) using Supelcosil^®^ LC-18-T RP column (4.6 × 250 mm, flow rate 1.0 mL min^−1^) with a linear gradient of methanol from 0 to 50% (*v*/*v*) in 0.05 M ammonium acetate (pH 5.9) with UV detection at 254 nm. The high-resolution mass spectrometry values were recorded with Micromass Q-TOF Premier using positive electro-spray ionization (HRMS-ESI). The NMR spectra were recorded by Agilent 600 MHz DDR2. 

The purity of the intermediates (a and b) was confirmed by mass spectrometry ([M-H]flow rate and [M+H]^+^ ESI). And, for the final compounds **3** and **4**, characterization was performed by HRMS and NMR.

Synthetic procedure:

N2-(prop-2-yn) -7-benzylguanosine 5′-monophosphate (**a**)

N2-(prop-2-yn)-GMP (1 eq.) was dissolved in 0.14 mL anhydrous DMSO, and benzyl bromide (5 eq.) was added. The reaction mixtures were stirred at room temperature for 3.5 h. Then, water (3 mL) was added, and the solution was extracted four times with diethyl ether (4 × 4 mL). The aqueous phases were purified on DEAE-Sephadex A-25 using a 0–1.0 M linear gradient of TEAB. Product (**a**) was obtained at a yield of 72% and characterized by MS ((ES+) m/z: (M+H)^+^:492.15; (ES−) m/z: (M-H)^−^: 490.05, calculated for C_20_H_23_N_5_O_8_P_1_^+^: 492.13).

N2-{1-[2-(2,6-dimethoxyphenoxy)propyl]-1H-1,2,3-triazol-4-yl}methylene-7-benzylguanosine 5′-monophosphate (**3**)

The final product **3** was obtained by performing click reactions according to the procedure described below (general procedure for the “click” reaction) on the intermediate compound (**a**) and was characterized by NMR and HRMS: 

3.2 mg (0.0044 mmoL), 15.7%, ammonium salt; ^1^H NMR (400 MHz, DMSO-d6): δ 8.08 (s, 1H, H8), 8.03 (s, 1H, triazole), 7.59 (d, 2H, Ph), 7.30–7.25 (m, 4H, Ph), 6.96–6.93 (m, 2H, Ph), 6.60 (d, 1H, H-1′), 5.60–5.56 (m, 2H, CH2benzyl), 4.52–4.50 (m, 3H, H-2′,NHCH2), 4.24–4.21 (m, 2H CH2CH2triazole), 4.13–4.10 (m, 2H, H-3′,H-4′), 3.83–3.81 (m, 2H, -CH2-O-Ph), 3.71 (s, 6H, CH3-O-Ph), 2.11–2.07 (m, 2H, CH2CH2CH2-OPh); ^31^P NMR (162 MHz, DMSO-d6) δ −2.92, HRMS(ES+) m/z: (M+H)+:729.23981, calculated for C_31_H_38_N_8_O_11_P_1_^+^: 729.22392.

P1-N2-(prop-2-yn)-7-benzylguanosine-P3-guanosine 5′,5′-triphosphate (**b**)

To dissolved P-imidazolide of guanosine 5′-diphosphate (im-GDP) (2 eq.) in anhydrous DMF, the intermediate compound (**a**) obtained earlier (1 eq.) and anhydrous ZnCl_2_ (10 eq.) were added, and then the solution was stirred vigorously for 24 h at RT. The reaction was quenched by adding an aqueous solution of EDTA in disodium salt (73 mg/1 mL). The resulting dinucleotide intermediate product was isolated from the mixture on DEAD-Sephadex gradient elution 0–1.0 M TEAB. The product was dissolved in H_2_O and was lyophilized, obtaining a fine white powder. Product (**b**) was obtained at a yield of 41% and characterized by MS ((ES+) m/z: (M+H)^+^:917.27; (ES−) m/z: (M-H)^−^:915.23, calculated for C_30_H_36_N_10_O_18_P_3_^+^: 917.14).

**P1-N2-{1-[2-(2,6-dimethoxyphenoxy)propyl]-1H-1,2,3-triazol-4-yl}methylene-7-benzylguanosine-P3-guanosine 5′,5′-triphosphate (4)**

The final product **4** was obtained by performing click reactions according to the procedure described below (general procedure for the “click” reaction) on the intermediate compound (**b**) and was characterized by NMR and HRMS:

3.8 mg (0.0032 mmoL), 29.1%, ammonium salt; ^1^H NMR (400 MHz, DMSO-d6) N2bz7G: δ 9.41 (s, 1H, H8), 8.03 (s, 1H, triazole), 7.56 (d, 2H, Ph), 7.41–7.35 (m, 4H, Ph), 6.57–6.55 (m, 2H, Ph), 6.18 (d, 1H, H-1′), 5.57–5.52 (m, 2H, CH2benzyl), 4.71–4.69 (m, 1H, H-2′), 4.65–4.56 (m, 2H with 3H, CH2CH2triazole), 4.54–4.51 (m, 1H, H-3′), 4.45–4.,39 (m, 1H with 2H, H-4′), 4.35–4.26 (m, 2H z 4H, 5H’, 5H’’), 3.86–3.73 (m, 2H, -CH2-O-Ph), 3.60 (s, 6H, CH3-O-Ph), 2.30–2.26 (m, 2H, CH2CH2CH2-OPh)4; G: δ 8.09 (s, 1H, H8), 5.80 (d, 1H, H-1′), 4.65–4.56 (m, 1H with 3H, H-2′), 4.50–4.47 (m, 1H, H-3′),), 4.45–4.39 (m, 1H with 2H, H-4′), 4.35–4.26 (m, 2H with 4H, 5H′, 5H″), ^31^P NMR (162 MHz, DMSO-d6) δ -14.70 (2P, Pα,γ), −26.07 (1P, Pβ); HRMS(ES+) m/z: (M+H)+:1154.25308, calculated for C_41_H_51_N_13_O_21_P_3_^+^: 1154.25298.

**General procedure for the “click” reaction**

Two solutions were prepared: Solution 1. 100 mg CuSO_4_ × 5 H_2_O in 10 mL of water; Solution 2. 10 mg of sodium ascorbate in 200 μL of water. To the suspension of (1eq.) of mononucleotide (**a**) or dinucleotide (**b**) in anhydrous DMSO, solution 1 (80 μL), TBTA (2.2 mg), solution 2 (38 μL), and 2-(3-azidopropoxy)-1,3-dimethoxybenzene (2 eq.) were added and stirred for 4 h at RT. Next, the reaction mixtures were terminated by the addition of EDTA (73 mg/1 mL). The final product **3** was purified on a reversed-phase HPLC column, and product **4** was purified by ion-exchange chromatography on a DEAE-Sephadex A-25 column using a linear 0–1 M TEAB gradient to produce compounds as TEA salts. The final dinucleotide was additionally purified on a reversed-phase HPLC column. Compounds were lyophilized to yield **3** (15.7%) and **4** (29.1%). The ass spectra of compounds (**a** and **b**) and both the high-resolution mass spectra and NMR spectra for compounds **2** and **3** are attached in the Supplementary Materials.

### 3.3. Translation Inhibition in RRL

Then, 2 mM stock solutions of compounds **3** and **4** in 10% DMSO were prepared, and their concentration was double-checked spectrophotometrically in a 0.1 M phosphate buffer pH 6.0 and calculated as an average of two readings: at 258 nm (extinction coefficient 11400) and 282 nm (extinction coefficient 7340) (as for the m^7^GMP analogues [14]). Stock solutions were subsequently used for serial dilutions (in 10% DMSO). As control compounds, m^7^GMP, m^7^GTP, m^7^GpppG, and bn^7^GMP were used as solutions in 10% DMSO. Experiments of the inhibition of the cap-dependent translation in rabbit reticulocyte lysate (Flexi RRL System, Promega) were performed as previously described [30]. Briefly, 56% of RRL lysate (batch with a 2 mM concentration of endogenous Mg^2+^), supplemented with magnesium acetate (1.2 mM), potassium acetate (200 mM), an amino acid mixture (0.01 mM), and a RiboLock RNAse inhibitor (0.32 units/μL) was preincubated for 60 min at 30 °C. Next, 1.25 μL of the tested compound (as a solution in 10% DMSO) and m_2_^7,3′O^GpppG-capped luciferase mRNA (to a final concentration of 5 ng/μL) was added to the reaction, and the translation mixture (12.5 μL final volume) was incubated for an additional 60 min. As a reference, reactions with DMSO (1.25 μL of 10% solution) were prepared. The reactions were stopped by chilling on ice, and the luciferase activity was determined in a Glomax luminometer (Promega). The IC_50_ values were obtained by fitting to the experimental data a theoretical curve that describes the extent of inhibition as a function of competitive inhibitor (e.g., cap analogue) concentration [24].

### 3.4. Thermal Stability Assay

The thermal stability of human full-length eIF4E (heIF4E) was analyzed using differential scanning fluorimetry (DSF), similar to that described previously for murine eIF4E [30]. heIF4E was expressed and purified as described earlier [33]. The assay sample (20 μL) contained 5× SYPRO Orange (Sigma Aldrich, St. Louis, MO, USA) and 4 μM of heIF4E (final concentration) in HEPES/KOH with 100 mM KCl, 0.5 mM EDTA, and 1 mM DTT (pH 7.2). The tested compounds, as 10× stocks in 10% DMSO, were added to the final concentration at a range from 1 μM to 200 μM. DMSO at a 1% final concentration was then added to the control reactions without any compounds. A CFX96 Real-Time PCR (Bio-Rad) was used to increase the temperature starting from 25 °C to 95 °C, with the temperature ramp at 0.5 °C/30 s, and the fluorescence intensity (FRET channel) was measured at each step. The melting temperature (Tm) was determined as the minimum of the first negative derivative with the built-in CFX Manager Software (Bio-Rad).

## 4. Conclusions

Modified monophosphates at the N2 position show significant translational inhibition compared to both m^7^GMP and m^7^GTP, as evidenced by numerous previous studies. On the other hand, the introduction of a benzyl modification at the N7 position resulted in a less definitive and somewhat experimentally dependent effect on inhibition compared to m^7^GMP, but bn^7^GMP (**1**) is a flagship example of cap analogues that have been used to prepare various types of pro-drugs. In our current study, we combined the concepts of modifying both of these positions. We synthesized doubly substituted analogues with aromatic groups at the N2 and N7 positions of the cap structure: a benzyl substituent at the N7 position and a (1-(3-(2,6-dimethoxyphenoxy)propyl)-1H-1,2,3-triazol-4-yl)methyl at the N2 position (m^7^GMP modified in this way showed excellent inhibitory properties). The results obtained from the biological tests of such doubly modified guanosine monophosphate indicate that the introduction of the N7-benzyl group has little effect on the inhibitory properties of the monophosphate having a substituent in the N2 position of guanosine. However, it should be taken into account that the presence of an additional hydrophobic group could have a negative impact on the solubility of the compound. Perhaps the solution would be to introduce substituents at the N2 position of the compound, which, when combined with bn7, would not reduce the solubility. However, an analogous double modification at the N2 and N7 positions is clearly advantageous for the dinucleotide. This may be related to the fact that in the case of dinucleotides, solubility is not a problem due to the presence of a triphosphate bridge. Therefore, introducing two modifications to the compound simultaneously at the N2 and N7 positions may be an effective way to improve the inhibitory effect of dinucleotides, suggesting their potential as a promising avenue for drug development in cancer therapy.

## Figures and Tables

**Table 1:** Inhibition of ARCA-mRNA luciferase translation in RRL system.

	IC_50_^DMSO^ ± SE [μM]	Inhibition Relative to m^7^GMP IC_50_^DMSO^ (m^7^GMP)/ IC_50_^DMSO^ (Cap Analogue)	Inhibition Relative to m^7^GpppG IC_50_^DMSO^ (m^7^GpppG)/ IC_50_^DMSO^ (Cap Analogue)	IC_50_^WATER^ ± SE [μM]
m^7^GMP	19.22 ± 3.31	1.00	0.23	>50
m^7^GTP	1.09 ± 0.14	17.60	4.08	2.30 ± 0.20
m^7^GpppG	4.45 ± 0.61	4.30	1.00	8.60 ± 0.70
bz^7^GMP (1)	10.86 ± 1.41	1.77	0.41	-
(4-(diOCH_3_-bn)-tz-CH_2_)_2_m^7^GMP (2)	1.80 ± 0.21	10.67	2.47	2.00 ± 0.10
(4-(diOCH_3_-bn)-tz-CH_2_)_2_bn^7^GMP (3)	2.70 ± 0.30	7.11	1.64	-
(4-(diOCH_3_-bn)-tz-CH_2_)_2_bn^7^GpppG (4)	0.24 ± 0.02	80.08	18.54	-

**Table 2:** Summary of binding affinity (appK) to heIF4E of newly synthesized doubly modified mononucleotide compounds.

	appK ± SE [μM] ^a^	Adj. R^2^	Relative Change in Affinity to m^7^GMP
m^7^GMP	24.45 ± 2.89	0.995	1
m^7^GTP	3.15 ± 0.84	0.985	7.8
m^7^GpppG	11.35 ± 1.09	0.975	2.2
bz^7^GMP (1)	31.10 ± 4.48	0.990	0.8
(4-(diOCH_3_-bn)-tz-CH_2_)_2_m^7^GMP (2)	13.28 ± 3.45	0.982	1.8
(4-(diOCH_3_-bn)-tz-CH_2_)_2_bn^7^GMP (3)	14.71 ± 1.14	0.994	1.7
(4-(diOCH_3_-bn)-tz-CH_2_)_2_bn^7^GpppG (4)	3.58 ± 1.09	0.971	6.8

^a^ *app*.K (μM)—apparent affinity was calculated based on the ΔTm change in heIF4E in the presence of an increasing concentration of the analyzed ligand. The Tm values were calculated using CDpal software, the obtained ΔTm data were plotted versus the corresponding concentration (in μM) of the analyzed compound (Figure 1), and *app*.Kd was calculated by fitting the single site ligand binding model function [32] using Origin Pro software. The *app*.K values presented here correspond to three independent experiments.

## Data Availability

All relevant data is included in the article and the Supplementary Material. Any further questions can be directed to the respective authors, as indicated in the article.

## Supplementary Materials

The following supporting information can be downloaded at https://www.mdpi.com/article/10.3390/ph17050632/s1, Compounds characterization (the NMR and HRMS spectra).
