# Supplementary material for: The Synergistic Effect of N2 and N7 Modifications on the Inhibitory Efficacy of mRNA Cap Analogues
Source: Pharmaceuticals (Basel). 2024 May 14;17(5):632. doi: 10.3390/ph17050632 (PMC11123931; doi:10.3390/ph17050632)
Supplement: Supplementary file 1 [file pharmaceuticals-17-00632-s001.zip › pharmaceuticals-2979608-supplementary.pdf]

## Supplementary Materials

# The Synergistic Effect of N2 and N7 Modifications on the Inhibitory Efficacy of mRNA Cap Analogues

Karol Kurpiejewski[1], Karolina Piecyk[1], Maciej Lukaszewicz[2], Karol Kamel[3], Kazimierz Chmurski[1], Sebastian Kmiecik[4] and Marzena Jankowska-Anyszka[1*]

[1]Faculty of Chemistry, University of Warsaw, 02-093 Warsaw, Poland
[2]Division of Biophysics, Institute of Experimental Physics, University of Warsaw, 02-093 Warsaw, Poland
[3]Institute of Bioorganic Chemistry, Polish Academy of Sciences, 61-704 Poznan, Poland
[4]Biological and Chemical Research Centre, Faculty of Chemistry, University of Warsaw, 02-089 Warsaw, Poland

*** Correspondence:** marzena@chem.uw.edu.pl, m.jankowska-an@uw.edu.pl

## The intermediate products

### N2-(prop-2-yn)-7-benzylguanosine 5'-monophosphate (a)

MS (ES+ and ES-)

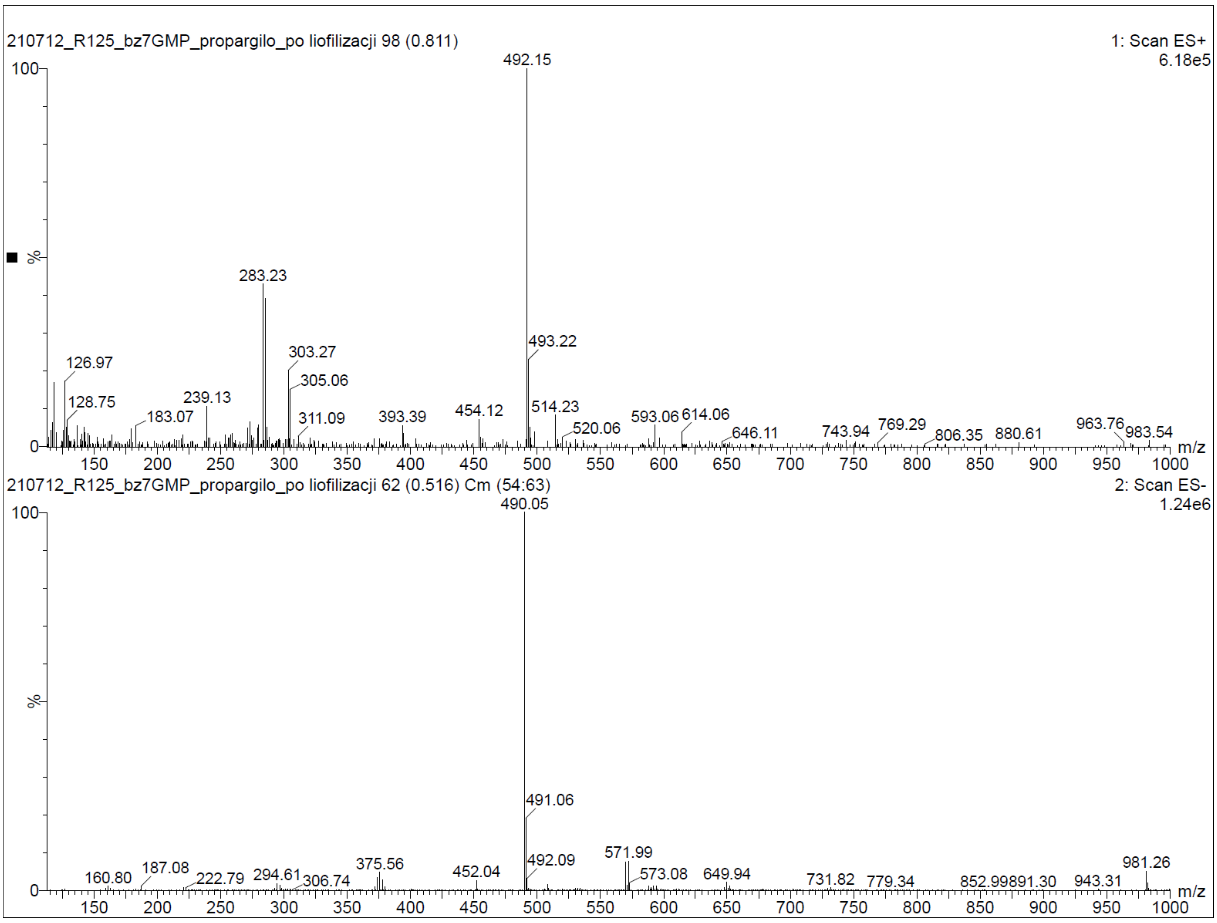

**P1-N2-(prop-2-yn)-7-benzylguanosine-P3-guanosine 5',5'-triphosphate (b)**

MS (ES+ and ES-)

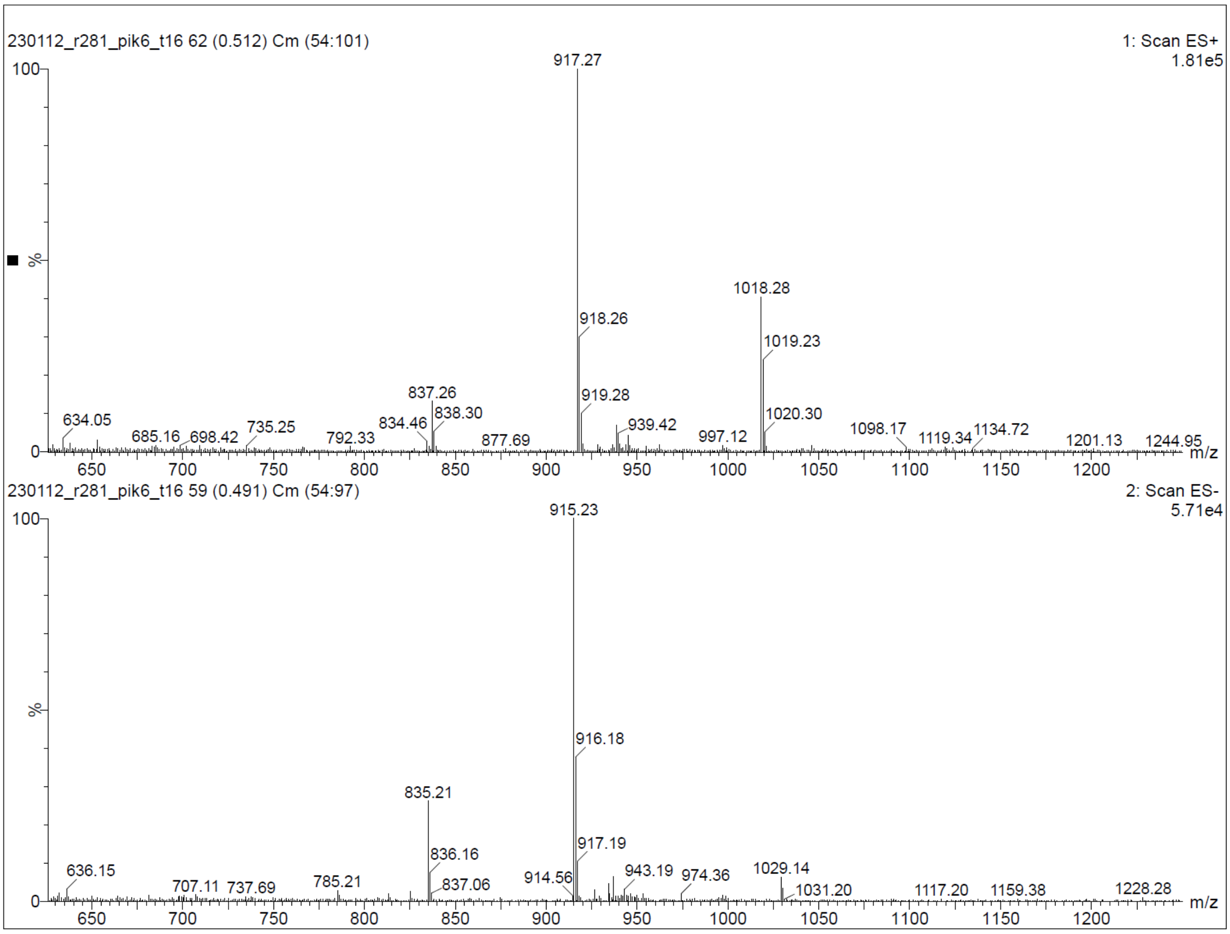

The final products:

**N2-{1-[2-(2,6-dimethoxyphenoxy)propyl]-1H-1,2,3-triazol-4-yl}methylene-7-benzylguanosine 5'-monophosphate (3)**

$^1$H NMR

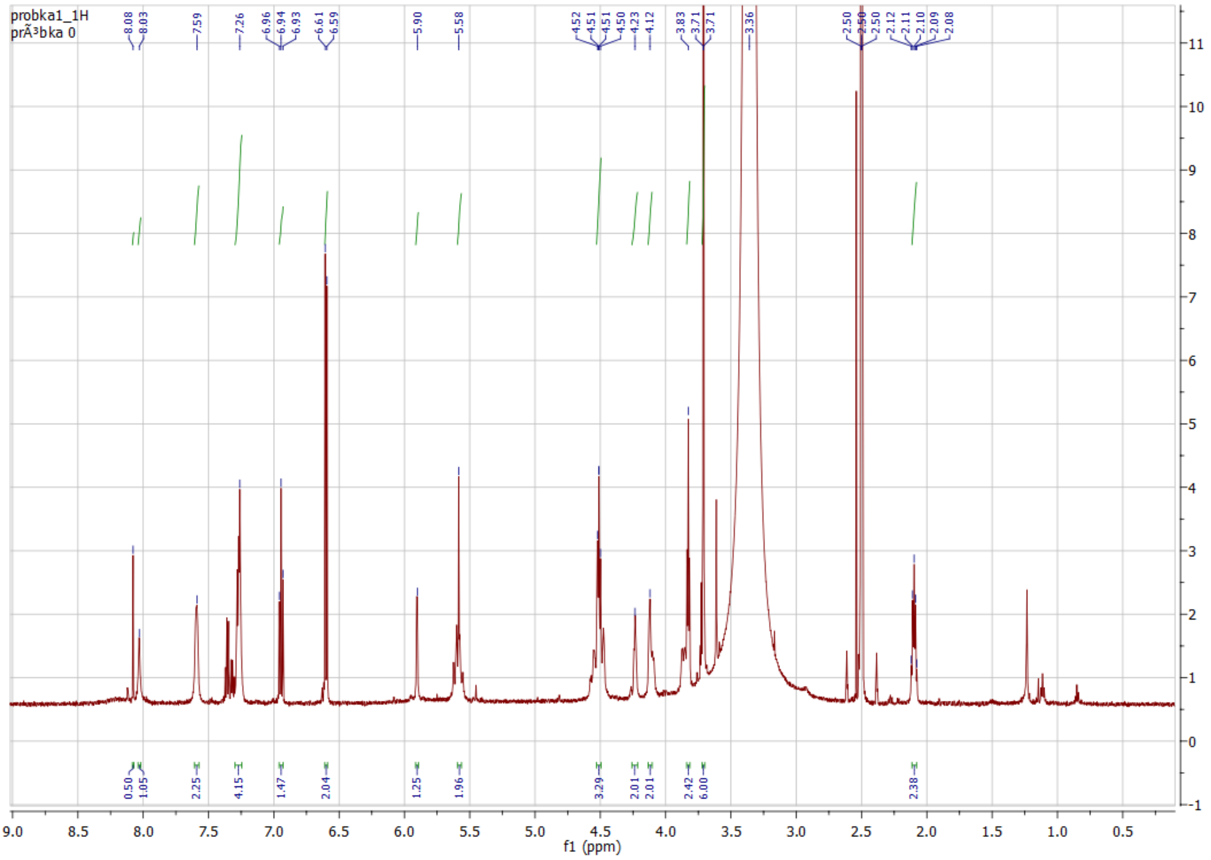

## $^{31}$P NMR

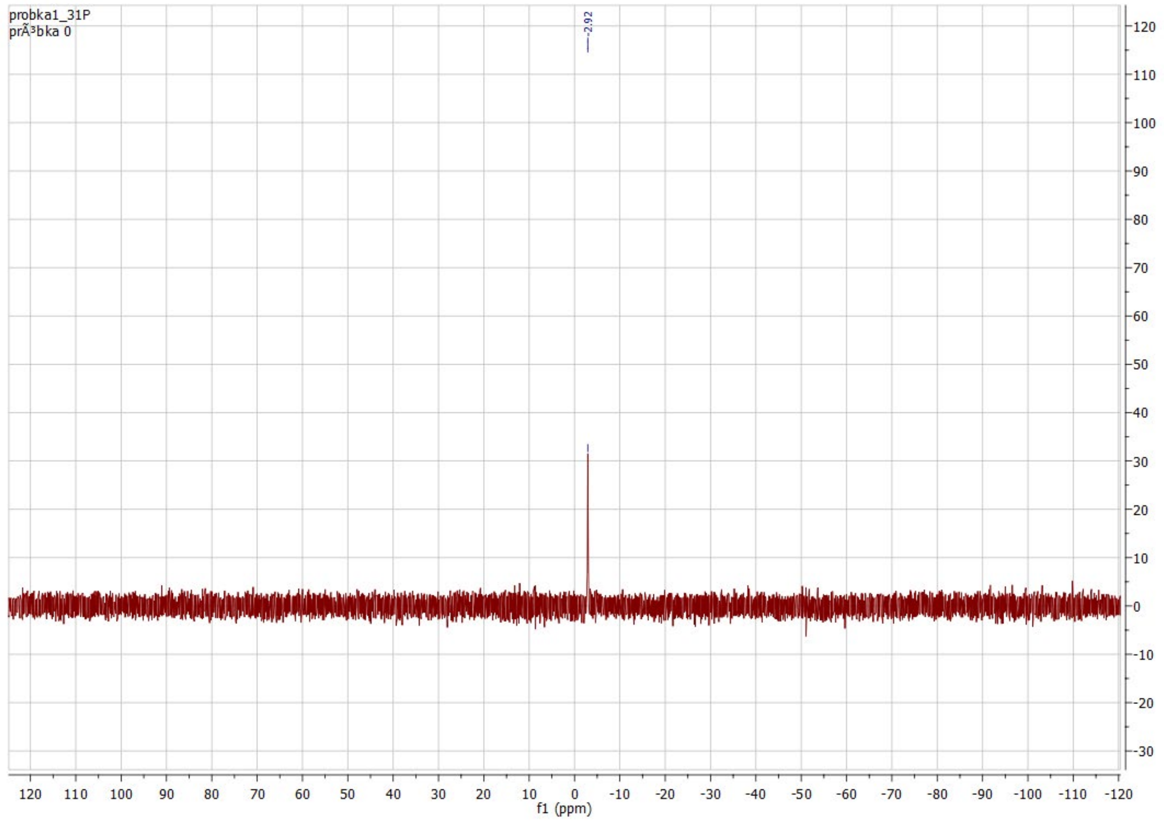


## HRMS (ES+)

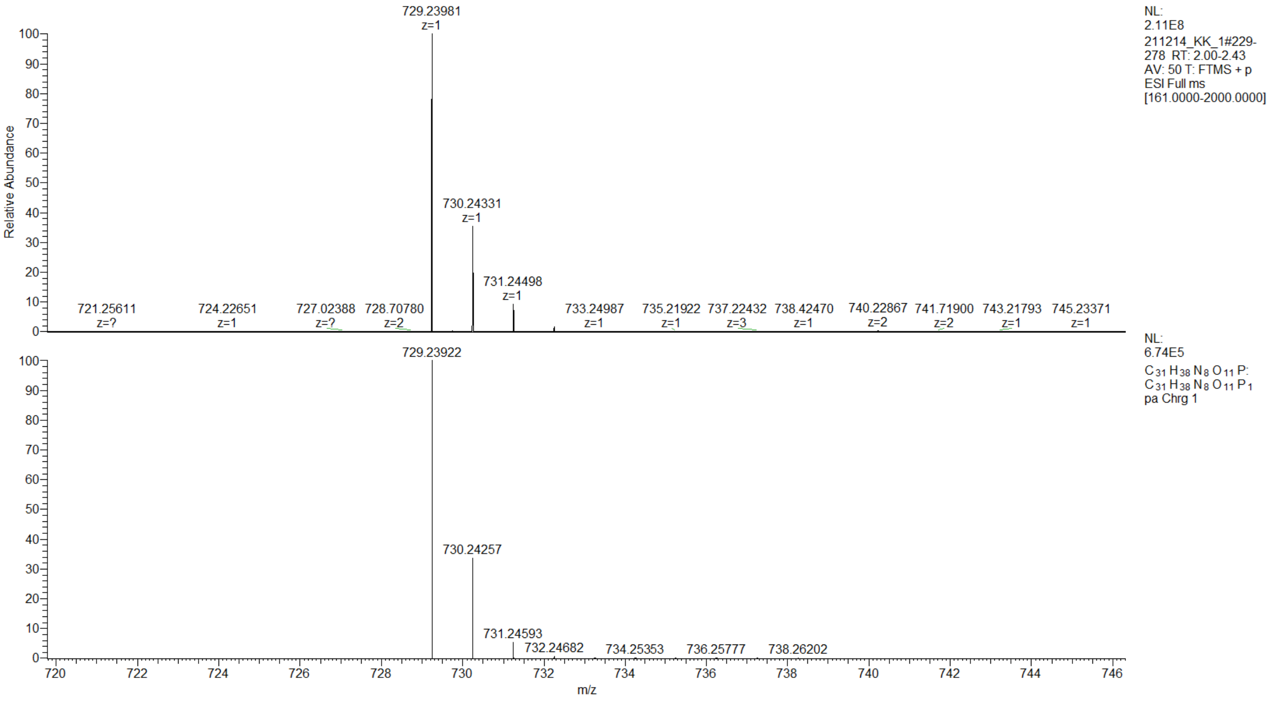

**P1-N2-{1-[2-(2,6-dimethoxyphenoxy)propyl]-1H-1,2,3-triazol-4-yl}methylene-7-benzylguanosine-P3-guanosine 5’,5’-triphosphate (4)**

$^{1}$H NMR

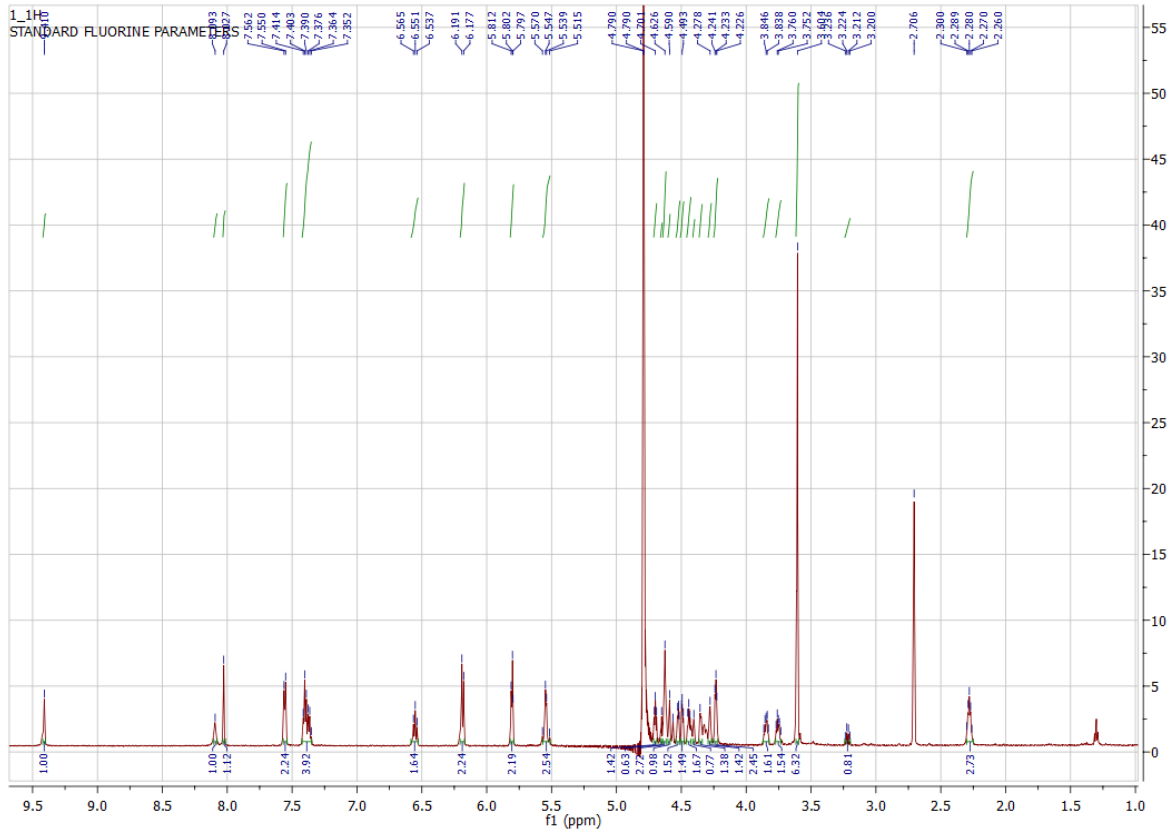


$^{31}$P NMR

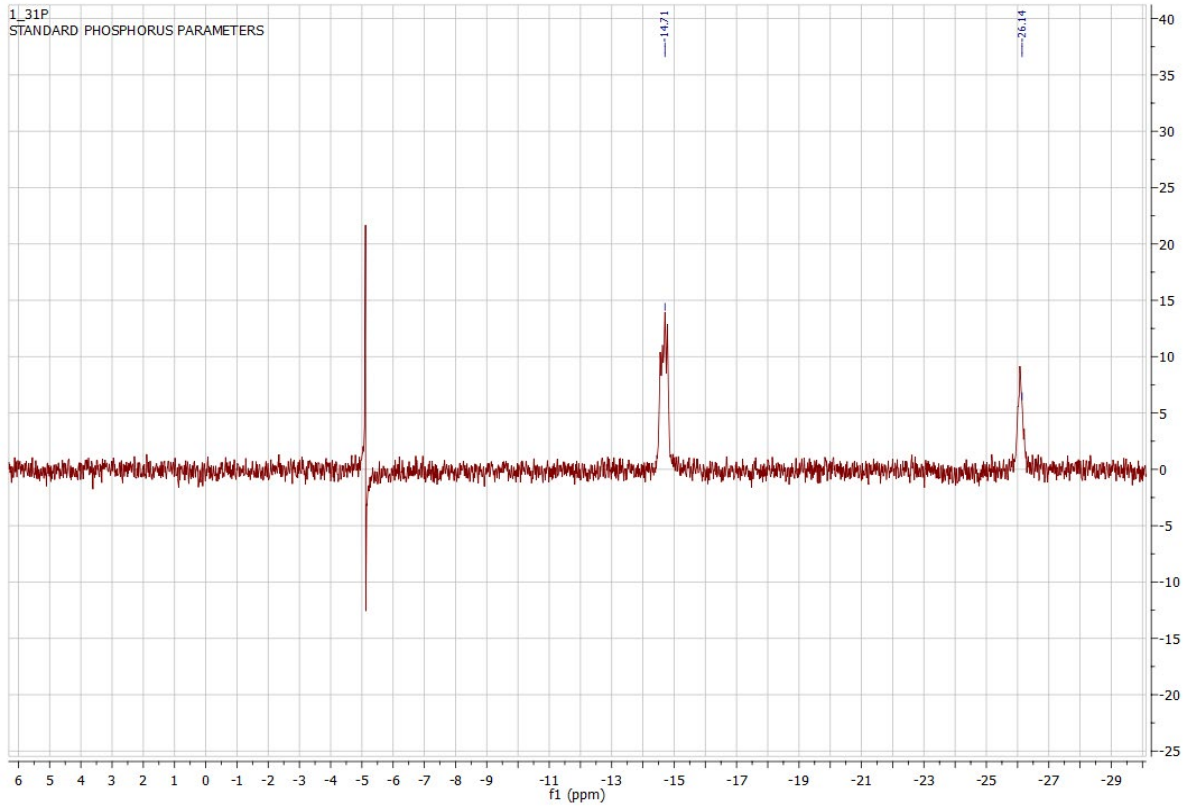

1_31P
STANDARD PHOSPHORUS PARAMETERS
-14.71
-26.14
f1 (ppm)


HRMS (ES+)

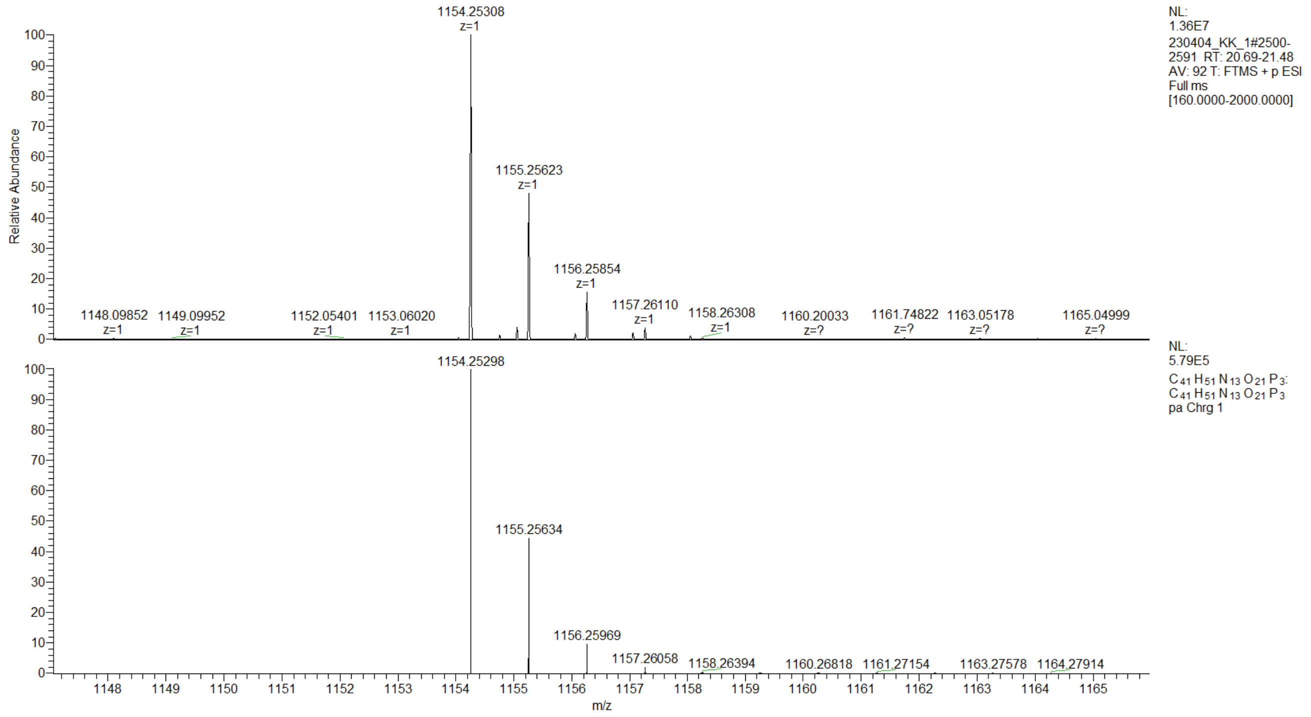

1154.25308
z=1
1155.25623
z=1
1156.25854
z=1
1157.26110
z=1
1158.26308
z=1
1148.09852
z=1
1149.09952
z=1
1152.05401
z=1
1153.06020
z=1
1160.20033
z=?
1161.74822
z=?
1163.05178
z=?
1165.04999
z=?
NL:
1.36E7
230404_KK_1#2500-2591 RT: 20.69-21.48
AV: 92 T: FTMS + p ESI Full ms [160.0000-2000.0000]
Relative Abundance
1154.25298
1155.25634
1156.25969
1157.26058
1158.26394
1160.26818
1161.27154
1163.27578
1164.27914
NL:
5.79E5
C41 H51 N13 O21 P3:
C41 H51 N13 O21 P3
pa Chrg 1
m/z
